# Advances in locoregional surgical treatment of *de-novo* stage IV breast cancer: a review

**DOI:** 10.3389/fonc.2026.1831950

**Published:** 2026-09-07

**Authors:** Shushu Liu, Mancheng Yu, Zirui Ke, Jingru Wang, Wei Zhong, Wei Wang

**Affiliations:** Breast cancer center, Hubei Cancer Hospital, Tongji Medical College, Huazhong University of Science and Technology, National key clinical specialty discipline construction program, Hubei Provincial Clinical Research Center for Breast Cancer, Wuhan Clinical Research Center for Breast Cancer, Wuhan, China

**Keywords:** bone-only metastases, de-novo stage IV breast cancer, locoregional surgery, primary tumor resection, prospective study

## Abstract

**Purpose:**

Breast cancer is the most common malignancy among women worldwide. A notable subset of patients presents with *de-novo* stage IV breast cancer at initial diagnosis, meaning metastatic lesions are already present. These patients typically receive primary systemic therapy aimed at palliative care, with locoregional treatment remaining controversial. Although numerous retrospective studies have suggested that resection of the primary tumor may improve survival, prospective randomized trials have not demonstrated a clear benefit, and routine surgical intervention is not generally advocated. This paper aims to review and discuss the research progress regarding locoregional surgical treatment for *de-novo* stage IV breast cancer.

**Methodology:**

Recent retrospective and prospective studies on locoregional surgery for *de-novo* stage IV breast cancer were searched, with a focus on prospective randomized controlled trials (RCTs). The collected evidence was reviewed to evaluate the role of local surgery in this patient population.

**Results:**

Most retrospective studies and meta-analyses report that primary breast surgery is associated with improved prognosis when stage IV disease is first diagnosed. However, significant selection bias exists in these data. Women who undergo surgery tend to be younger, have smaller primary tumors, fewer comorbidities, and lower metastatic burden—particularly fewer visceral metastases. They are also more likely to receive higher-quality overall care. These factors suggest that surgeons preferentially select patients with a better expected prognosis for surgery. In contrast, prospective randomized trials have consistently failed to demonstrate that locoregional surgery provides additional survival benefits beyond systemic therapy alone. Consequently, routine locoregional surgical treatment is not recommended for most patients with *de-novo* stage IV breast cancer. Nevertheless, a notable exception exists for patients with bone-only metastases.

**Conclusion:**

Although retrospective analyses support a survival advantage from locoregional surgery in *de-novo* stage IV breast cancer, prospective RCTs have not confirmed a benefit for the general patient population. However, locoregional surgery does appear to offer survival benefits for patients with bone-only metastases. Further prospective clinical trial data are certainly needed to better define the value of locoregional surgery in *de-novo* stage IV breast cancer.

## Highlights

- Retrospective studies suggest a survival benefit from locoregional surgery, but results are limited by selection bias.
- Prospective randomized trials show no overall survival benefit of locoregional surgery in unselected patients.
- Patients with bone-only metastases may derive survival benefit from locoregional surgical treatment.
- ″ Routine locoregional surgery is not recommended for *de-novo* stage IV breast cancer based on current evidence.

## 1 Introduction

As the most common malignant tumor for women, breast cancer is the malignant tumor with the highest incidence among women and the fifth highest mortality rate, and the vast majority of breast cancer patients who die are advanced patients (1, 2). Despite the fact that the global breast cancer mortality rate has been decreasing since 1990s, approximately 6% of breast cancer patients are still diagnosed with stage IV breast cancer (3). Traditionally speaking, stage IV breast cancer has been considered an incurable disease, and the goals of its treatment are to prolong life, reduce or prevent symptoms, and improve quality of life (4). But it still remains controversial whether locoregional surgical treatment is able to provide a survival benefit to patients with *de-novo* stage IV breast cancer (5).

Currently, NCCN guidelines promote the individualized decision-making for local therapy for stage IV tumors; however, there are growing evidences on how active local therapy can improve survival in other stage IV malignant tumors (6, 7). The 2020 edition of the NCCN guidelines states that (1) surgical treatment can be directed at the primary site when the local tumor can be completely removed and the metastatic condition is not life-threatening, and (2) surgery is applied only to patients who have a need for relief of painful symptoms such as skin ulcers, bleeding, cauliflower-like growths, infections, and pain after systemic therapy (8). In the past two decades, much attention has been focused on the role of locoregional surgical treatment for *de-novo* stage IV breast cancer, but there is no certain agreement on conclusions (9, 10). Therefore, this paper will review the relevant literature both at home and abroad to discuss the research progress of locoregional surgical treatment for *de-novo* stage IV breast cancer.

Relevant studies were identified by searching PubMed, Web of Science, Scopus and Google scholar using keywords including “*de-novo* stage IV breast cancer,” “locoregional surgery,” and “primary tumor resection,” focusing on retrospective studies, prospective randomized trials, and meta-analyses published from 2005 to 2023. This review aims to provide a nuanced synthesis by highlighting the subgroup of patients with bone-only metastases who may benefit from locoregional surgery, incorporating the most recent prospective trial data, and offering a structured summary table for clinical reference.

## 2 Impact of locoregional surgical treatment for *de-novo* stage IV breast cancer on survival

### 2.1 Retrospective studies

Over the past two decades, several retrospective single-institution and population-based studies have shown that the resection of the primary tumor of *de-novo* stage IV breast cancer can obtain survival advantages and thus improve survival rate. Khan et al. conducted their analysis on the largest data from the National Cancer Database (NCDB) from 1990 to 1993, which evaluated 1, 6032 patients with stage IV breast cancer. 57% of these patients underwent surgery for the primary breast tumor (segmental mastectomy accounts for 38.3% and mastectomy accounts for 61.7%), compared with 43% who had no surgical intervention (11).The overall survival rate was 24.9% during the three-year observation, with 17.3% without surgery, 27.7% with segmental mastectomy and 31.8% with mastectomy (*P* <.0001). Despite the different types of surgery, negative surgical margins were associated with the improvement on 3-year survival rate. Independent prognostic factors included surgical removal of the primary tumor, status of surgical margins, receiving systemic therapy, number of metastatic sites and type of metastatic disease. Similarly, Gnerlich et al. found that among 9,734 patients with stage IV breast cancer, the median survival rate of patients who had surgery has improved in comparison with those who did not have surgery (12). Rapiti et al. evaluated 300 patients with stage IV breast cancer, whose overall survival rate was 16%, 27% of which were operated with negative margins, 16% with positive margins, 12% with margins and unknown margins, and 12% without surgery (13). This approach can reduce the mortality risk from negative margins by 40% compared with patients who did not undergo surgery or who underwent surgery with positive margins. In addition, only bone metastases were associated with improved survival to surgery compared to other metastatic sites.

In a 2012 meta-analysis published by Petrelli et al, 15 paper met the study criteria and were retrieved from the total of 7, 071 papers dating from 2002 to 2012, the result of which suggests that the survival benefit of primary tumor in stage IV breast cancer was more favorable for patients who underwent surgical treatment (14). Another 2016 meta-analysis selected 19 papers eligible for study criteria from 1, 628 relevant papers and displayed that the hazard ratio (HR) of the overall survival (OS) for patients treated with surgery was 0.63 (95% confidence interval (CI), 0.58-0.70), suggesting that undergoing surgery could reduce the risk of mortality by 37% (15). Xie Yuxin et al. from Xijing Hospital in China published a retrospective study in 2017, which enrolled 223 patients with primary tumor in IV breast cancer diagnosed from 1999 to 2014, with a median follow-up visit time of 24 months (16). Patients were divided into 177 cases (79.4%) in the primary tumor surgery group and 46 cases (20.6%) in the non-surgical group.

All of these retrospective studies show that prognosis can be improved by primary surgical resection of the breast with stage IV disease. However, there are other sources of bias in the retrospective data and the meta-analyses based on the relevant data. Women who undergo surgery tend to be younger, have smaller tumors, fewer comorbidities, lower metastatic burden, fewer visceral metastases, and are more likely to receive better care, which may thus imply that surgeons tend to select patients who are likely to survive longer (17, 18). Despite the fact that all studies have attempted to control these biases using multiple regression, the amount of unknown bias may still change even after these known biases are statistically adjusted.

Meanwhile, surgery may disrupt the original balance of the tumor in the body, increasing the risk of metastasis. It has been shown through animal experiments that surgery is thought to remove the growth inhibitory factor of the primary lesion during the removal of the tumor of the primary lesion, which can promote tumor angiogenesis and thereby result in tumor metastasis (19).

Hence, surgeons should be extremely cautious in applying these data to their treatment strategies, as the value of locoregional surgical treatment of primary tumors in *de-novo* stage IV breast cancer apparently has yet to be proven.

### 2.2 Prospective studies

#### 2.2.1 The Indian TATA study

The first prospective randomized controlled trial (NCT00193778) was held in 2005 at the Tata Memorial Centre in Mumbai, India. It recruited 350 patients with *de-novo* metastatic breast cancer and an expected survival of 1 year (20). Preoperative chemotherapy consisted of anthracyclines in combination with or without paclitaxel, and patients were later randomly allocated to a surgical (with radiotherapy) versus non-surgical group (without radiotherapy), which is also the control group. The main outcomes were overall survival and progression-free survival respectively. The non-surgical group had 20.5% overall survival rate after 5 years, while the surgical group had 19.2% (HR 1.04; 95% CI, 0.80-1.34). The risk of local progression-free survival was lower in the surgical group, but that of distal progression-free survival was much higher. In subgroup analyses (menopausal status, visceral vs bone metastases, >3 vs 1-3 metastatic sites and hormone receptor/HER2 status), no significant differences were observed as for survival rate. However, this clinical trial had some limitations. First, due to the late diagnosis, most patients already had clinical symptoms at the time of diagnosis; second, 31% of the patients were HER2-positive, but due to financial constraints, only 15% received targeted therapy, whereas none did so in the local therapy group; and third, only some patients received paclitaxel-based chemotherapy. These aforesaid factors resulted in a median OS of only about 20 months in both groups, which was lower than that of developed countries. Therefore, the study could not accurately assess the impact of surgery on the overall prognosis of patients receiving standard chemotherapy and targeted therapy.

#### 2.2.2 MF07-01 trial

Some argue that all patients with *de-novo* stage IV breast cancer should be randomly allocated before they receive systemic therapy, and those who were assigned to the surgical group receive locoregional treatment in advance, which is a more effective test for the locoregional surgical therapy in the metastatic condition. However, this also implies that locoregional surgical treatment is offered to patients who may not respond to certain systemic treatments at distant metastatic sites. Therefore, the Turkish MF07-01 trial conducted from 2008 to 2012 was based on this logic of design. All of the subjects who have not undergo treatment were randomly assigned into groups and received either systemic therapy alone or surgical treatment of the primary tumor (with or without axillary lymph node dissection) and subsequent radiotherapy (if indicated), plus systemic therapy. All patients received hormonal therapy as needed, and HER2-positive patients received trastuzumab (21). The trial enrolled a total of 278 patients, with its primary study objective being overall survival and secondary objective including morbidity, local progression or recurrence, and improvement in quality of life. The trial was reported at SABCS 2013 with a median follow-up period of 18 months and 31% mortality rate. No significant survival benefit was observed at the time of reporting, with a median overall survival of 46 months in the initial surgical treatment group and 42 months in the initial systemic treatment group (HR 0.76; 95% CI 0.49-1.16; *P* = 0.20). By analyzing the nonpredetermined subgroup, the outcomes were similar in most patients: surgery prolonged survival in patients with single bone metastasis, but shortened survival in patients with multiple liver or lung metastases. The rate of locoregional progression was substantially lower in the initial surgical treatment group than in the initial systemic treatment group (0.7% vs. 3.6%). And then, after 5 years, the improvement in overall survival was 41.6% in the surgical group versus 24.4% in the systemic treatment group. The median survival of the surgery group was 46 months, 9 months longer than the 37 months of the systemic therapy group. Subgroups with higher postoperative overall survival included ER-positive, HER2-negative, isolated bone metastases and age less than 55 years. Due to the differences between groups, the surgical group had fewer patients with triple-negative subtypes and visceral metastases, and their local progression were only 1% compared with 11% in the systemic therapy group. In this trial, patients underwent surgery prior to systemic therapy, thus limiting the assessment of therapeutic response and the prediction of the outcome.

#### 2.2.3 ECOG-ACRIN 2108

On account of the inability of the Indian TATA study to accurately assess the impact of surgery on the overall prognosis of patients receiving standard chemotherapy and targeted therapies, the Turkish MF07-01 trial limited the assessment of treatment response to preoperative systemic therapy (22, 23). Therefore, ECOG E2108, a phase III prospective multicenter study, evaluated the role of neoadjuvant systemic therapy as the initial treatment for stage IV breast cancer patients whose condition, after receiving such therapy, were stable or improved and continued to undergo surgery (24). This study was based on the patients with *de-novo* stage IV breast cancer and their characteristics of tumor and applied optimal systemic therapy (OST) accordingly, and patients who were progression-free within 4-8 months after receiving OST were further selected and randomly categorized into the continued systemic therapy group and the locoregional therapy (LRT) group, and patients’ progression-free survival (PFS) and OS were evaluated. A total of 390 patients were treated with OST, and of all the 256 eligible patients, 131 were randomized to continued OST alone and the rest 125 received OST combined with LRT. There were 121 deaths and 43 patients with locoregional progression (range: 0-91) after a median follow-up of 59 months. OS rate was neither significantly different between the two groups [3-year OS rate was 68.4% in the OST combined with LRT group and 67.9% in the OST alone group, *P* = 0.63, HR = 1.09, 90% CI: 0.80-1.49], and nor was there any significant difference in PFS (*P* = 0.40) between the two groups. Patients in the OST alone group had a significantly higher rate of locoregional recurrence or progression than patients in the OST combined with LRT group (25.6% vs 10.2%, *P* = 0.003). The health-related quality of life (HRQOL) was measured by FACT-B Trial Outcome Index of the OST combined with LRT group was significantly worse than the OST alone group (60% completion, p=0.01) 18 months after random allocation into groups, but no difference was observed at 6 months (74% completion) or 30 months (56% completion). Early locoregional therapy did not improve the survival of patients with initial stage IV breast cancer. Although the risk of locoregional disease progression was 2.5-fold higher without LRT, IPT patients who received LRT did not have improved HRQOL. Indeed, this trial also had limitations and lacked multivariate analysis. One of its randomized postoperative criteria was “complete resection without positive margins,” which was achieved in 87 out of 109 patients, and approximately 20% of LRT patients still had positive surgical margins. In addition, there was still no clear and detailed information on the extent of axillary surgery and radiotherapy, and it lacked subgroup analysis according to the site of metastasis. Although 38% patients had bone metastases alone, subgroup analysis was not conducted. Likewise, organ-specific comparisons between groups were not performed. However, the ECOG-ACRIN 2108 study provided important data in terms of local control, which matters significantly as for patients with oligometastases, especially those with bone metastases alone.

#### 2.2.4 BOMET MF 14-01

This study aims to evaluate the role of locoregional therapy (LRT) in patients with *de-novo* stage IV breast cancer with bone only metastases (BOM). This prospective registry-based study, the multicenter BOMET MF 14-01, began in May 2014 with patients with *de-novo* stage IV bone-only metastases divided into two groups: patients receiving systemic therapy (ST group) and patients receiving LRT (LRT group) (25). Patients who received LRT were further divided into two groups: ST after LRT (LRT + ST group) and ST before LRT (ST + LRT group). The study included 505 patients, with 240 (47.5%) patients in the ST group and 265 (52.5%) in the LRT group. At a median follow-up of 34 months, 113 patients (26.3%) died, of which 85 (35.4%) were in the ST group and 28 (10.5%) in the LRT group. Local progression was observed in 39 cases (16.2%) in the ST group and 18 cases (6.7%) in the LRT group (*P* = 0.001). The risk of mortality was 60% lower in the LRT group compared with the ST group (HR 0.40, 95% CI 0.30-0.54, *P* < 0.0001). LRT was found to prolong survival and reduce the rate of locoregional recurrence at a median 3-year follow-up. In patients with *de-novo* stage IV breast cancer with bone-only metastases, locoregional surgical treatment at diagnosis or after systemic therapy had a similar survival benefit compared with systemic therapy alone.

#### 2.2.5 JCOG 1017

The Japanese JCOG 1017 program enrolled 410 patients with *de-novo* metastatic breast cancer to compare the efficacy of primary tumor resection plus systemic therapy versus systemic therapy alone (26). After 3 months of systemic therapy, patients without disease progression were randomly assigned to undergo surgery or to continue systemic therapy (without radiotherapy), with the primary objective being overall survival. The secondary study objective was the rate of locoregional recurrence and local control, as well as the effect on distant metastases after resection of the primary tumor. At the 59^th^ Annual Meeting of the American Society of Clinical Oncology (ASCO) in 2023, the Japan Clinical Ontology Group presented the results of the JCOG1017 phase III randomized controlled clinical trial, which enrolled 570 patients for the first time during the period of May 11, 2011, to May 31, 2018. Among the enrolled patients, 407 eligible patients were randomly assigned to either the systemic therapy alone group (N = 205) or the primary resection group (N = 202), and the patients’ characteristics were balanced between the two groups. The median overall survival time (MST) of patients in the randomized group was 70 months, with 221 deaths. The difference in OS was not statistically significant (HR 0.857, 95% CI: 0.686 to 1.072, *P* = 0.3129). The MST was 68.7 months in the systemic therapy alone group and 74.9 months in group B. The proportions of patients with progression-free metastatic sites were respectively 81.5% and 67.3% in the systemic therapy alone group and primary resection group (*P* = 0.0014). Local recurrence-free survival (LRFS) was significantly longer in the primary resection group than in the systemic therapy alone group (median LRFS: 19.6 vs 62.9 months; HR 0.415, 95% CI: 0.327 to 0.527, *P* < 0.0001). In the primary resection group, patients with incomplete resection of the primary lesion had worse outcomes than those with complete resection (94.5 vs. 60.7 months, HR 1.971, 95% CI: 1.161 to 3.347; *P* = 0.012)). In subgroup analyses, primary tumor resection (PTR) improved survival in patients with ER-positive tumors, premenopausal status or single-organ metastases.

#### 2.2.6 Other case studies

Three prospective studies with similar inclusion criteria including pathologically confirmed *de-novo* stage IV breast cancer and receipt of first-line systemic therapy are summarized. In the TBCRC013 study, all the enrolled 112 patients with *de-novo* stage IV breast cancer who received first-line systemic therapy were divided into a surgical group and systemic therapy group based on whether or not the patients underwent surgery (27). The survival rates were 77% and 76% respectively, but surgery did not result in any further survival benefit. The study states that patients with primary tumors in *de-novo* stage IV breast cancer should not choose surgical treatment, except in clinical studies. The POSYTIVE trial in Austria, of which the actual enrollment had only 90 patients, was divided into surgical and non-surgical groups, with 45 patients in each group (28). The OS was 34.6 months in the surgical group and 54.8 months in the non-surgical group, with no significant difference between the groups. The study suggested that there was a trend toward worse prognosis for patients in the surgical group, and that it markedly reduced patients’ quality of life. In 2018 ASCO Annual Meeting, Yu et al. from Sun Yat-Sen University reported that 189 (53.6%) of the 353 patients enrolled in the study underwent surgery for primary lesion, and 164 (46.4%) were not performed with surgery, with a median follow-up time of 22.1 months, and the 5-year survival rates of patients in the surgical and non-surgical group were respectively 62.4% and 60.3%, and there was no statistically significant difference in OS between the groups, which indicates that for surgical treatment for primary lesion failed to bring survival benefit to patients (29).

#### 2.2.7 Meta-analyses and systematic reviews

Several meta-analyses and systematic reviews have synthesized the available evidence on locoregional surgical treatment for *de-novo* stage IV breast cancer. A 2012 meta-analysis by Petrelli and Barni included 15 retrospective studies and reported that primary tumor resection was associated with improved overall survival (hazard ratio 0.69, 95% confidence interval not reported, *P* < 0.00001) (30). However, the authors acknowledged that all included studies were retrospective and subject to significant selection bias. A 2025 systematic review and meta-analysis by Sun et al. included 8 prospective studies (2029 patients) and found that locoregional surgery significantly improved locoregional progression-free survival (HR 0.36, 95% CI 0.14-0.95, *P* = 0.04) and overall survival in ER/PR-positive patients (HR 0.77, 95% CI 0.55-0.93, *P* = 0.01), but not in unselected patients (31). Collectively, these meta-analyses highlight the need for more prospective randomized trials to definitively determine the role of locoregional surgery.

## 3 Discussion

A summary of key studies, including their design, study period, sample size, outcomes, and limitations, is provided in **Table 1**. The current research has not reached consensus on any clear reports or consistent conclusions on whether locoregional surgical treatment improves survival in patients with primary stage IV breast cancer (32, 33). Although retrospective data suggest that locoregional therapy may provide a survival advantage for women with metastatic breast cancer, it should not be routinely recommended that *de-novo* stage IV breast cancer patients undergo locoregional surgery until additional, unbiased data are available, as the bias in the retrospective data includes surgical treatment in younger women with smaller tumors, single metastases, and less visceral disease, as well as the fact that randomized trials of prospective studies have not demonstrated a further survival benefit conferred by surgery (31). In particular, if distant metastatic lesions are not well controlled, and the patients’ survival time may not be long enough to make locoregional primary lesions an issue, or if both local and distant metastatic lesions are well controlled, in this case, the locoregional primary lesion is likely to be well controlled throughout the patient’s lifetime (34). Surgery may be an alternative approach for patients whose distant metastases are already controlled but local primary lesions are still progressing, but for patients with *de-novo* stage IV breast cancer with bone-only metastases, locoregional surgical treatment at the time of diagnosis or after systemic therapy can offer certain survival benefit (35).

**Table 1:** Summary of key studies on locoregional surgical treatment for *de-novo* stage IV breast cancer.

Study (year)	Design	Study period	Population (N)	Primary survival outcomes	Locoregional control	Subgroup findings	Methodological limitations
Retrospective studies							
Khan et al. (2017) (11)	Retrospective (NCDB)	1990-1993	16,032	3-year OS improved with surgery	Negative margins improved OS	Not reported	Selection bias; younger/healthier patients selected
Gnerlich et al. (2008) (12)	Retrospective (SEER)	1988-2003	9,734	Median OS improved with surgery	Not reported	Not reported	Selection bias; no adjustment for metastatic burden
Rapiti et al. (2006) (13)	Retrospective	Not specified	300	Negative margins reduced mortality by 40%	Bone-only mets showed better survival	Not reported	Single institution; small sample
Xie et al. (2017) (16)	Retrospective	1999-2014	223	Median follow-up 24 months	Not reported	Not reported	Single center in China; small sample
Prospective RCTs							
Badwe et al. (2015) (20) (Indian TATA)	RCT	2005-2010	350	5-year OS: 20.5% vs. 19.2%; HR 1.04	Lower local progression in surgery group	No significant differences by subgroup	Low HER2-targeted therapy use (15%); late diagnosis
Soran et al. (2021) (23) (MF07-01)	RCT	2008-2012	278	5-year OS: 41.6% vs. 24.4%; median OS: 46 vs. 37 months	Locoregional progression: 0.7% vs. 3.6%	Benefit in ER+, HER2-, bone-only mets, age <55	Surgery prior to systemic therapy; group imbalance
Khan et al. (2022) (41) (E2108)	RCT	2011-2015	390 (131 randomized)	3-year OS: 68.4% vs. 67.9%; HR 1.09	Locoregional progression: 10.2% vs. 25.6%	No subgroup analysis by metastatic site	20% positive margins; no subgroup for bone-only
Shien et al. (2023) (26) (JCOG1017)	RCT	2011-2018	570 (407 randomized)	Median OS: 74.9 vs. 68.7 months; HR 0.857	LRFS: median 62.9 vs. 19.6 months	Benefit in ER+, premenopausal, single-organ mets	No radiotherapy in control group
Fitzal et al. (2019) (28) (POSYTIVE/ABCSG-28)	RCT (terminated early)	2011-2015	90	OS: 34.6 vs. 54.8 months	Not reported	Not reported	Poor recruitment; statistically underpowered
Prospective registry/cohort studies							
Soran et al. (2021) (25) (BOMET MF14-01)	Prospective registry	2014-2018	505	Mortality risk 60% lower with LRT (HR 0.40)	Local progression: 6.7% vs. 16.2%	Similar benefit with LRT before or after ST	Non-randomized; selection bias
King et al. (2016) (27) (TBCRC013)	Prospective cohort	2005-2012	112	Survival: 77% vs. 76%	Not reported	Not reported	Small sample; no significant difference
Yu et al. (2021) (29)	Prospective cohort	2000-2014	353	5-year OS: 62.4% vs. 60.3%	Not reported	Not reported	Retrospective analysis of prospective data

OS, overall survival; RCT, randomized controlled trial; LRT, locoregional therapy; ST, systemic therapy; LRFS, locoregional recurrence-free survival; HR, hazard ratio; ER, estrogen receptor; HER2, human epidermal growth factor receptor 2; NCDB, national cancer database; SEER, surveillance, epidemiology, and end results; mets, metastases.

The HER2CLIMB-05 trial showed that adding tucatinib to first-line maintenance therapy significantly improved progression-free survival in HER2-positive metastatic breast cancer, with 70% of enrolled patients having *de-novo* stage IV disease (36). The FEATURE trial validated FDG-PET/CT for assessing treatment response in patients with bone-only or bone-dominant metastases, a subgroup that may benefit from locoregional surgery (37).

In low- and middle-income countries where access to novel systemic therapies is limited, retrospective data suggest that upfront locoregional surgery remains a common practice (38). A prospective cohort from Malawi reported that 26% of breast cancer patients presented with *de-novo* stage IV disease, and a case series from North Macedonia showed 100% 36-month survival after upfront surgical resection in luminal patients with bone-only metastases (38, 39).

Hence, the role of locoregional surgery in *de-novo* stage IV breast cancer is gradually declining, and locoregional surgical treatment needs to take into consideration the benefits and opinions of all parties involved case by case, and it also requires more individualized treatment with a multidisciplinary treatment (MDT) (40). In the meantime, further multicenter, multifaceted, multilevel, and multidimensional data from prospective clinical trials are also expected for the treatment of *de-novo* stage IV breast cancer with locoregional surgery (41).

Given the limitations of available evidence, no strong clinical recommendations can be made at present. Routine locoregional surgery is not recommended for unselected patients with *de-novo* stage IV breast cancer, but may be considered for those with bone-only metastases or symptomatic local progression. Treatment decisions should be individualized through multidisciplinary discussion, taking into account patient characteristics, metastatic burden, and disease biology. Further multicenter, prospective clinical trial data are certainly needed on the value of locoregional surgery for *de-novo* stage IV breast cancer. Future trials should focus on patient selection by metastatic site and tumor biology, especially in bone-only and oligometastatic subgroups, and should also include patient-centered endpoints such as standardized quality-of-life assessments in their protocols.
